# Deciphering the mechanistic landscape of notch signaling in autoimmune pathogenesis

**DOI:** 10.1007/s10238-026-02272-7

**Published:** 2026-07-31

**Authors:** Ruiyuan Zhang, Shenghao Tu, Weiji Lin, Liang Han, Xin Ba, Jiahui Yan, Yuyao Yang, Yifei Li, Tingting Li, Chennan Li, Guanghuan Li, Zhe Chen, Kai Qin, Ying Huang, Yu Wang, Yao Huang

**Affiliations:** Department of Integrated Traditional Chinese and Western Medicine, Tongji Hospital, Tongji Medical College, Huazhong University of Science and Technology, Wuhan, Hubei China

**Keywords:** Notch signaling pathway, Autoimmune diseases, Rheumatoid arthritis, Research progress, Review

## Abstract

Autoimmune diseases are a group of disorders with multiple etiologies that disrupt normal immunological function, leading to the immune system mistakenly attacking the body’s own tissues. The Notch signaling pathway is a conserved pathway that is in part responsible for multiple cellular behaviors, including proliferation, differentiation, development, and death, through direct cell-to-cell communication. The expression and activation of Notch signaling pathway proteins are aberrantly regulated in autoimmune diseases in a manner that is closely related to their onset and development. We reviewed the functioning of the Notch signaling pathway in several autoimmune disorders, including rheumatoid arthritis (RA), systemic lupus erythematosus (SLE), primary Sjögren’s syndrome (pSS), juvenile idiopathic arthritis (JIA), idiopathic thrombocytopenic purpura (ITP), multiple sclerosis (MS), autoimmune vasculitis, autoimmune hepatitis (AIH), autoimmune thyroid disease (AITD), autoimmune kidney diseases and type 1 diabetes mellitus (T1DM). We specifically sought to explore the involvement of the Notch signaling pathway in the occurrence and progression of RA to provide ideas and approaches for developing new therapeutic strategies for this autoimmune disease.

**Graphical Abstract:**

**Figure:**
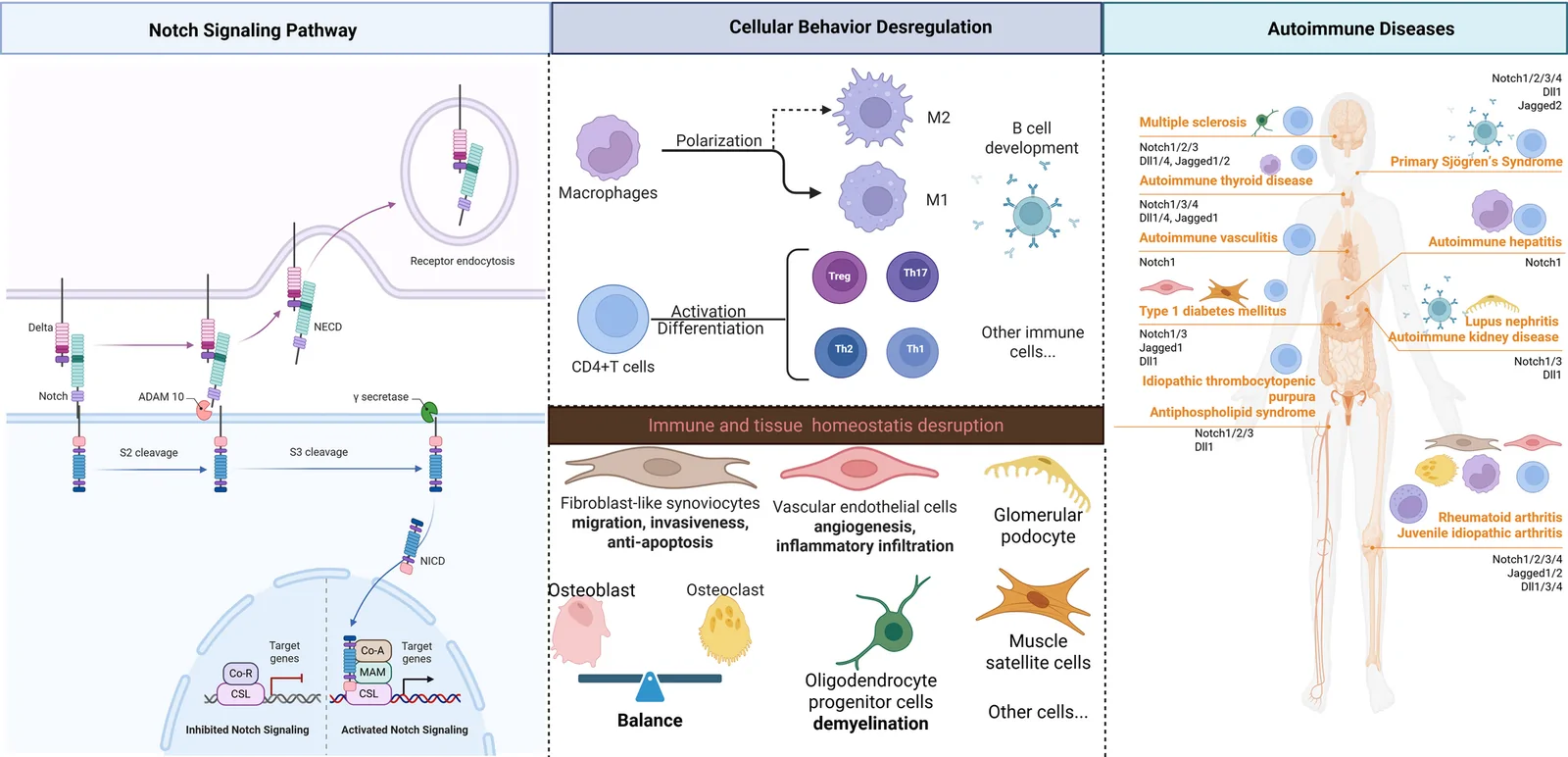

## Introduction

The Notch signaling pathway is a highly conserved cellular behavior-regulating pathway present in various organisms that mediates signal transduction between adjacent cells through receptor–ligand binding. Autoimmune diseases are a diverse group of diseases that occur when the body’s immune system attacks its own tissues because of the influence of genetic or environmental factors. This disruption of self-tolerance or immune cell regulation leads to ongoing immune responses in the presence of the body’s own antigens, causing damage or dysfunction in the affected tissues. Autoimmune diseases include common conditions such as rheumatoid arthritis (RA), systemic lupus erythematosus (SLE), and multiple sclerosis (MS) and impose an exceptionally heavy burden on health care systems and patient quality of life worldwide. Owing to a critical lack of effective strategies for reversing tissue injury, failing to timely halt disease progression in its early stages can lead to permanent and debilitating functional loss within target organs.

Recent research has provided increasing evidence for the role of the Notch signaling pathway in the development of autoimmune disorders. Overactivation of the Notch pathway is strongly linked to the proliferation, differentiation, and dysfunction of immune cells in autoimmune disorders. Furthermore, the Notch pathway plays a role in the aberrant regulation of injured self-tissues and interacts with other signaling pathways, crucial factors in the onset and progression of autoimmune disorders. By mediating intercellular communication and intersecting cross-talk with alternative signaling networks, the Notch pathway precisely modulates the developmental fates and behavioral phenotypes of both immune populations and target-organ stromal cells, thereby shaping the strength, pattern, and progression of autoimmune responses. Increasing evidence indicates that individual Notch receptors and ligands perform a highly sophisticated division of labor that is affected by the specific microenvironment and the respective disease pathological stage. Because disruption of this carefully balanced network directly drives the pathological diversity of autoimmune responses, a comprehensive examination and elucidation of the distinct functions of the components of each pathway remains essential to enable precise and individualized targeted therapeutics for autoimmune disorders. While preclinical evidence supports the therapeutic potential of targeting the Notch pathway in the treatment of autoimmune disorders, these findings require validation in clinical trials, as clinical investigations concerning even the most extensively studied γ-secretase inhibitors remain limited. Nevertheless, the central role of this pathway in regulating immune cell development and behavior suggests its vast therapeutic potential. Alongside advancements in precision and individualized drug design, modulating Notch signaling activity is a highly promising research and translational direction for autoimmune therapy.

## Composition and activation of the Notch signaling pathway

In mammals, the Notch signaling pathway consists of four Notch receptors, five Notch ligands, C-promoter binding protein-1 (CSL), and various Notch signaling regulatory molecules. The four Notch receptors—Notch1/2/3/4—and the five Notch ligands—Jagged1/2 and Dll1/3/4—are membrane proteins that are expressed on the cell membrane surface [1]. Genes regulated by the Notch pathway through transcription include mainly the Hes family and Hey family of genes, themselves members of the basic helix-loop-helix (bHLH) transcription repressor family [1].

The classical Notch pathway involves three key enzymatic cleavage steps. Notch receptors are typically synthesized in an inactive single-peptide precursor form in the endoplasmic reticulum. The complete Notch receptor includes the extracellular region, the transmembrane region, and the intracellular domain (NICD). The peptide that contains the Notch receptor sequences is first transmitted to the Golgi apparatus, where it is cleaved by the furin-inverting enzyme, forming mature Notch receptor heterodimers that are transported to the cell surface; this process is referred to as the first cleavage [2]. After the mature Notch heterodimer extracellular domains bind to ligands on the cell membrane, the heterodimers undergo a conformational change, followed by a second cleavage mediated by the ADAM metalloproteinase, which removes the extracellular portion of the Notch receptor [2]. Following the second cleavage, the remaining part undergoes a crucial third cleavage by γ-secretase, producing a soluble NICD fragment that enters the nucleus. Upon entering the nucleus, the NICD binds to the transcription protein CSL, converting the “coexpressor complex” to the “coactivator complex”, thereby activating downstream gene expression [2].

In addition to the classical activation pathway, the Notch pathway can also alter cellular behavior through nonclassical mechanisms. Notch receptors can undergo endocytosis and activate target genes without binding to CSL, directly triggering downstream target gene transcription by interfering with other signaling pathways, even in the absence of ligand binding [1].

## Notch signaling pathway and rheumatoid arthritis (RA)

RA is a chronic autoimmune disease characterized by persistent inflammation of the synovium. RA has a high incidence, causes significant disability, and severely affects the quality of life of the affected patients. Its primary pathological features include synovial infiltration of macrophages and lymphocytes, abnormal proliferation of fibroblast-like synoviocytes (FLSs), and joint destruction. The pathogenesis of RA is intricate and involves dysregulation of multiple immune cells and synovial stromal cells, resulting in the formation of a sophisticated regulatory network. Environmental and genetic factors also play important roles in the onset of RA. Importantly, the Notch signaling pathway serves as a critical mediator in RA by regulating the activation, differentiation, and pathogenic behaviors of multiple immune cell subsets and synovial stromal cells, thereby contributing to chronic joint inflammation and destruction (Tables 1 and 2).

**Table 1:** Summary of clinical evidence of the involvement of the Notch signaling pathway in major autoimmune diseases

Disease types	Notch components	Sample sources	Clinical findings	Refs.
Rheumatoid arthritis	Notch4	peripheral nucleated blood cells	significant association between RA and Notch4 polymorphisms	[3]
	Jagged2, Dll4	Memory CD4 + T cells	rs874040(CC) allele skews memory T cells toward a pro-inflammatory phenotype	[4]
	Dll1, Jagged1, Jagged2	Peripheral Blood Mononuclear Cells (PBMCs)	hsa-miR155−3p was directly proportional to Dll1 and Jagged1, and MALAT1 was inversely proportional to Jagged2	[5]
	Notch1, Dll4	synovium	Notch1, Dll4, and Hrt1 expression were found in synovium	[6]
	Notch1,Nocth2	synovium	Strongly detected in RA synovium	[11]
	Notch3, Jagged1	synovium	Notch3 and Jagged1 are overexpressed in FLS	[12]
	Notch2, Notch3, Notch4	Th cells	Increased expression of Notch 2/3/4 in Th cells from active RA patients	[22]
	Notch3	CD14 + cells	miR−233 was upregulated in activated RA CD14 + cells, and correlated with reduced Notch3 expression	[28]
Systemic Lupus Erythematosus	Notch1	glomeruli	Notch1 pathway was overactivated in glomeruli of LN patients	[47]
	Notch3	glomeruli	Notch3 was overexpressed in glomeruli of LN patients	[48]
primary Sjogren’s syndrome	Notchs	PBMCs	Patients with higher serum IgG levels exhibited DNA methylation enrichment in Notch signaling	[53]
	Notch4	/	Notch4 is identified as pSS-related gene by GWAS in Chinese Han population	[54]
	Notch2	Salivary glands and tonsils from patients with pSS	Salivary glands and tonsils contained Notch2 mRNA	[55]
Juvenile idiopathic arthritis	Notch2, Jagged1	PBMCs from JIA children	The mRNA of Notch2 and Jagged1 were highly expressed in JIA samples, and downstream Hes1 mRNA expression was positively correlative with CD3 + T cell ratio	[59]
Idiopathic thrombocyto penicpurpura	Notch1	Peripheral blood	Notch1 and downstream Hes1 mRNA expression were upregulated in ITP	[60]
	Notch1, Notch3, Dll1	PBMCs from ITP patients	Notch1, Notch3 and Hes1 expression elevated, while Notch2 and Dll1 decreased in ITP patients	[61]
	Notch1	PBMCs from ITP patients	Notch1 expression is increased in ITP patients, and could not reach the level of healthy people after effective treatment	[62]
	Notch1	Peripheral blood	Notch1 mRNA were lower than those of normal control; the levels of CD4 + CD25+ CDl27low Treg postively correlated with Notch1 mRNA	[63]
	Notchs	CD4 + T lymphocytes	Differentially methylated CpG sites enriched in Notch signaling pathway	[64]
	Notch1	Bone marrow mononuclear cells from ITP patients	Notch1 gene showed significant differential methylation compared to control	[65]
	Notch1, Notch2, Notch3	PBMCs from ITP patients	The Notch1/2/3 mRNA of acute ITP were higher than that of chronic ITP or health control	[66]
Multiple sclerosis	Notch2	CD14 + monocytes	Notch2 expression on CD14 + monocytes was identified as baseline predictors of nADA development in MS patients treated with IFN-β	[67]
Grave’s disease	Notch4	/	Notch4 single-nucleotide polymorphism was significantly associated with young-age-onset GD	[89]
Hashimoto thyroiditis	Notch1, Dll4	PBMCs from HT patients	Notch1 and D114 mRNA levels were higher than health; Notchl and D114 mRNA levels were significantly positively correlated with the proportion of Th17 cells; Notchl and D114 mRNA levels were positively correlated with the serum TPOAb and TgAb titers	[90]

**Table 2:** Summary of experimental evidence of the involvement of the Notch signaling pathway in major autoimmune diseases

Disease types	Evidence category	Notch components	Crosstalk	Models/Species	Phenotype	Refs.
Rheumatoid arthritis	In vitro	Notch1, Dll4	Notch1/HIF−1α	HDMEC; RA-FLS	Angiogenesis; invasiveness	[6]
	In vitro	Notch1	/	HMVEC; RA-FLS	Angiogenesis, glycolysis, invasiveness, pro-inflammation	[7]
	In vitro	Notch1, Notch3	HIF−1α/Notch1 HIF−1α/Notch3	RA-FLS	invasiveness, pro-angiogenesis	[8]
	In vitro	Notch1, Dll4	JAK2/STAT3/Notch1 JAK2/STAT3/Dll4	RA-FLS, RA synovium explants	Invasiveness, migration and cytokine production	[9]
	In vitro	Notch3	/	RA-FLS, HUVEC	Pannus formation	[10]
	In vitro	Notch2, Dll2, Dll3	/	RA-FLS	IL−6 secretion	[13]
	In vitro	Notch1	Notch1/NF-κB2	RA-FLS	proliferation	[14, 15]
	In vitro	Notch1, Notch4, Jagged2	/	RA-FLS	Proliferation, invasiveness	[16]
	In vitro	Notch1, Jagged1	Survivin/Notch1/Jagged1/Hes1	RA-FLS	Proliferation, pro-angiogenesis and apoptosis	[17]
	In vitro	Notch1	Notch1/VEGFR1/2	RA explants	Angiogenesis	[18]
	In vitro	Notch1, Notch4	Notch1/VEGF/Ang2	HMVEC	Invasiveness, angiogenesis	[19]
	In vivo, in vitro	Notch1	Notch1/NF-κB/H3K27 acetylation	Cre/Lox-induced conditional mutations of endothelial Notch in C57BL mice HUVEC	Upregulation of adhesion molecules	[21]
	In vivo	Notch1	miR−449a−5p/Notch1	CIA mice	Th17/Treg balance	[23]
	In vivo	Notch1	/	CIA and CAIA mice	Modulating the population and suppressive function of Treg cells	[24]
	In vivo	Notchs, Dll3	/	CIA mice	Inflammatory responses of Th1 and TH17 cells	[25]
	In vitro	Notch1, Dll1	/	Spleen mononuclear cells from Collagen-Ⅱ-immunized DBA/1J mice	Th1 and Th17 expansion	[26]
	In vitro	Notch3	Notch3/miR−223/AHR/ARNT	Macrophages (THP−1)	Pro-inflammatory cytokine expression	[28]
	In vivo, in vitro	Notch2, Dll1	/	CIA mice, macrophages from synovium of CIA mice	IL−6, GM-CSF, and MMP−3 production	[29]
	In vivo	Notch1	/	Hes1-GFP/TNF-Tg mice	M1 polarization and joint destruction	[30]
	In vivo, in vitro	Notchs	TNF/p52/RELB/NICD	mesenchymal stem cells from RA patients, TNF-tg mice	Osteoblast differentiation, inflammation-mediated bone loss	[31]
	In vitro	Notch2	IL−20/Notch2/NFATc1	BMDM from rats	Osteoclastogenesis	[32]
	In vivo, in vitro	Jagged1	TNF-α/RBPJ/Jagged1	Mice with myeloid/macrophage-specific deletion/expression of Jagged1; bone marrow derived macrophages (BMDM) from mice, human osteoclasts derived from CD14 + cells.	Inflammatory Osteoclastogenesis	[33]
	In vivo, in vitro	Notch1, Notch2, Dll1, Jagged1	/	BMDM from mice, human PBMC, K/BxN serum-induced arthritic mice, ovariectomized mice	The differential regulation of osteoclastogenesis by Notch2/Dll1 and Notch1/Jagged1	[34]
Systemic Lupus Erythematosus	In vivo	Notch1	Notch1/NLRP3	MRL/lpr mice	Podocyte injury	[47]
	In vivo	Notch3	YB−1G/Notch3	MRL/lpr mice	Enlarged lymphoid organs and aggravated nephritis	[48]
	In vivo	Notch1	/	BABL/C-lpr mice	Corticosteroid sensitivity	[49]
	In vitro	Notch1	miR−30a/Notch1	MPC5	Podocyte injury	[50]
	In vivo	Notchs, Dll1	/	MRL/lpr mice and nephrotoxic serum-induced LN models	B cell activation, renal ectopic lymphoid structure formation and renal injuries	[51]
Antiphospholipid syndrome	In vitro	Notchs	miR−326/Notchs	Monocytes isolated from PBMCs of APS patients	Monocyte activation and adhesion factor production	[52]
Primary Sjogren’s syndrome	In vivo	Notch2	Notch2/NF-κβ14	C57BL/6.NOD-Aec1Aec2 mice	The migration of marginal zone B lymphocytes to lacrimal glands	[56, 57]
	In vitro	Notch1, Notch3, Dll1, Jagged2	/	Submandibular gland protein-induced pSS rat models	Inhibition of Treg differentiation and Reduction in lung function	[58]
Idiopathic thrombocyto penicpurpura	In vitro	Notch1	/	Bone marrow mononuclear cells from ITP patients	Th1/Th2 differentiation	[65]
	In vitro	Notchs	Notch/AhR/IL22	CD4 + T cells from ITP patients	IL22 production and Th22 differentiation	[66]
	In vitro	Notch1	/	PBMCs from ITP patients	Th17/Treg imbalance	[67]
	In vitro	Notch1	lncRNA PVT1/PVT1/ubiquitination/Notch1	PBMCs cells from ITP patients	Th17/Treg imbalance	[68]
Multiple Sclerosis	In vivo	Notch1, Notch3	NF-κB/Notch	EAE mice	Inflammation	[70]
	In vivo, in vitro	Dll4	/	EAE mice, CD4 + T cells from splenocytes of mice	Myelin-specific Th2/Treg immune responses and impaired Th1/Th17 responses	[71]
	In vivo, in vitro	Notch1	IL−17/Notch1	Primary astrocytes from neonatal mice, gene edited EAE mice	Th17-induced inflammation and demyelination	[72]
	In vivo, in vitro	Notch1	lncRNA Gm13568/Notch1/STAT3	EAE mice, primary astrocytes	Inflammation, demyelination	[73]
	In vitro	Notch1	Contactin/Notch1/TIP30	Oligodendrocyte precursor cells	oligodendrocyte differentiation, remyelination	[74]
	In vivo, in vitro	Jagged1	BMP4/Notch signaling	Oligodendrocyte progenitors isolated from neonatal rats, cuprizone-induced demyelination mice	Differentiation of oligodendrocyte progenitor cells into mature oligodendrocytes	[76]
	In vivo	Notch1	/	Cuprizone induced acute demyelination mice	Oligodendrocyte differentiation, remyelination	[77]
	In vivo	Notch1	/	EAE mice	microglia/astrocyte activation, demyelination, CD4 + T cells infiltration in CNS	[78]
	In vivo, in vitro	Notch1	/	EAE mice, Notch1 knockout macrophages	Proinflammatory cytokine production	[79]
	In vivo	Jagged1, Dll1	/	EAE mice	Demyelination, remyelination	[80]
	In vivo, in vitro	Notch1, Notch2, Jagged2	Notch1/Smad3/IL9	EAE mice, CD4 + T cells from NICD1^fl/fl^, Notch1^fl/fl^, Notch2^fl/fl^ transgenic mice	Th9 development	[81]
Giant cell arteritis	In vivo, in vitro	Notchs	/	CD4 + T cells, human Artery-SCID Mouse Chimeras	The differentiation of vasculogenic T cells	[82]
Takayasu arteritis	In vitro	Notch1	Notch1/AMPK	CD4 + T cells from TAK patients	The maintaining of vasculogenic proinflammatory T cells	[83]
	In vivo, in vitro	Notch1	Notch1/mTORC1	CD4 + T cells from TAK patients, Human artery-NSG mouse chimeras	Th1 and Th17 differentiation	[84]
ANCA related crescentic glomerulonephritis	In vivo	Notchs	/	Nephrotoxic serum induced anti-murine glomerular basement membrane nephritis mice	Glomerular damage, podocyte injury	[85]
Membranous nephropathy	In vivo, in vitro	Notch1	/	Murine podocytes, SD rats	Renal function injury, renal tissue cell apoptosis, and podocytosis	[86]
Autoimmune hepatitis	In vitro	Notch1	miR−20b/Notch1	Macrophages (Raw264.7)	Hepatic fibrosis, inflammation	[87]
	In vivo, in vitro	Notchs	O-GlcNAc glycosylation of Notchs	Con A-induced AIH models established using GlcNAc glycosylation-deficient rats, CD4 + T cells from rats	Treg differentiation	[88]
Grave’s disease	In vitro	Notch1	miR−363/STAT4/HSPB1/Notch1	CD4 + T cells from GD patients	Treg cell dysfunction	[91]
Autoimmune thyroiditis	In vivo, in vitro	Notchs	Notch/ PI3K/AKT/mTORC1	Experimental autoimmune thyroiditis mice and splenic mononuclear cells from them	Th17 differentiation	[92]
	In vivo, in vitro	Notch3, Jagged1	OX40L/Jagged1/Notch3	Experimental autoimmune thyroiditis mice, CD4 + T cells and BMDM from mice	Treg expansion, production of IL−4 and IL−10	[93]
	In vivo	Notch1	/	Experimental autoimmune thyroiditis mice	Th17 differentiation	[94]
	In vivo	Notch1, Notch3, Jagged1, Dll1	/	High-iodine induced NOD.H−2h4 mice	M1/M2 polarization	[95]
Thyroid-associated ophthalmopathy	In vitro	Notchs	Serelaxin/Notch signaling	Primary orbital fibroblasts	Fibrosis	[96]
Type 1 Diabetes Mellitus	In vivo	Dll1	/	Akita mice	Muscle satellite cell injury, myogenic capacity decline	[97]
	In vivo, in vitro	Notch3, Jagged1	/	HUVEC, human coronary artery endothelial cells, STZ induced T1DM mice, Engineered tissues composed of microvessels	Arterio-venous specification, perivascular cell coverage	[98]
	In vivo	Notch3	/	Notch3-tg mice	The expansion and function of Tregs	[99]

### The Notch signaling pathway and genetic susceptibility to RA

Genetic variations and noncoding RNA dysregulation within the Notch signaling pathway significantly contribute to an individual’s susceptibility to RA through the altering of gene expression profiles and predisposing the individual to pathogenic phenotypes. At the genetic level, the Notch4 exon 3 polymorphism (C+1297T) is a prominent risk factor that is strongly associated with the susceptibility to RA [3]. The presence of the RA susceptibility locus rs874040 (CC) is associated with the upregulation of recombination signal-binding protein for immunoglobulin kappa J region (RBPJ) in memory CD4 + T cells [4]. Specifically, RBPJ, a transcriptional repressor, is converted into a transcriptional activator upon Notch pathway activation. When a Notch ligand binds a Notch receptor, the expression of RBPJ increases in a manner associated with the increased production of the inflammatory cytokines IL-9, IL-17 A and IFN-γ, reflecting a pathogenic T-cell phenotype [4]. Noncoding RNA networks can also modulate the Notch pathway; for example, downregulation of lncRNA MALAT1 coupled with upregulation of hsa-miR-155-3p disrupts standard signaling in RA, where increased expression of miR-155-3p is directly associated with increased expression of Dll1 and Jagged1 [5].

### The Notch signaling pathway and hypoxic environments in the synovium of individuals with RA

The hypoxic microenvironment of the synovium of individuals with RA acts as a potent upstream trigger of Notch signaling activation, subsequently modulating the behavior of joint destructive cell populations and synovial metabolic reprogramming. This pathogenic state drives excessive proliferation and greater oxygen consumption in FLSs, further exacerbating critical ischemic–hypoxic conditions within the joint cavity. Consistent with these pathophysiological observations in patients, an analysis of human RA synovial tissues by Gao and colleagues revealed that increased expression of NICD1 is positively associated with low oxygen partial pressure [6]. Similarly, Biniecka and colleagues reported that the hypoxic environment in RA patient-derived synovium leads to clinical hallmarks of mitochondrial dysfunction, including increased mitochondrial DNA mutation, elevated reactive oxygen species production, and altered glycolysis [7]. The results from subsequent experimental models provide definitive mechanistic evidence that the hypoxia-Notch network controls FLS invasion, cytokine release, and angiogenic sprouting. Chen and colleagues reported that high HIF-1α expression directly regulates the expression of the Notch1 and Notch3 genes under controlled hypoxic conditions [8]. Inhibition of Notch1/3 expression, in contrast, mitigates the invasion of RA-FLSs and the synovial angiogenesis induced by hypoxia, whereas the overexpression of NICD1/3 promotes these processes [8]. Subsequent investigations demonstrated that under hypoxic conditions, inhibition of JAK2 signaling in RA-FLSs specifically and effectively reduced the expression of Notch1, Dll4, and the downstream target genes Hrt-1/2 [9]. In vitro inhibition of glycolysis significantly mitigated the invasion, migration, and secretion of proinflammatory factors in FLSs and human umbilical vein endothelial cells (HUVECs) as well as synovial angiogenesis and was associated with the inhibition of HIF-1α, p-STAT3, and NICD1 activation [7, 9]. Finally, the results from experimental models developed by Wei and colleagues revealed that Notch3 orchestrates signaling cross-talk between sublining FLSs and vascular endothelial cells (VECs), which serves to guide the FLSs and facilitate the formation of new blood vessels [10].

### Notch signaling pathway and synovial stromal cells

Notch signaling plays a key role in shaping the pathogenic behavior of synovial stromal cells, primarily by shaping the aggressive phenotype of FLSs and promoting aberrant angiogenesis of VECs.

#### Notch signaling pathway and FLSs

Abnormal activation of the components of the Notch signaling pathway affects the proliferative, migratory, and invasive capacity of FLSs. Evidence from clinical specimens has consistently demonstrated that Notch signaling is dysregulated in RA. Chen and colleagues reported significantly upregulated NICD1/3 in the RA synovium [8]. Similarly, Western blot and immunohistochemical staining analyses revealed strongly positive expression of Notch1/2 in the RA synovium [11]. During the early stage of development of the synovium of infants, all Notch homologs exhibit very positive expression [11]. These expression patterns suggest that excessive Notch activation is linked to the synovial proliferation, migration, and invasion observed in RA patients. Furthermore, high-resolution profiling of RA-FLS subgroups revealed that the expression of Jagged1 and Notch3 are constitutively upregulated, whereas the expression of Jagged2, Dll1, Dll4, and Notch1 are relatively moderate under baseline clinical conditions [12]. This expression pattern reflects a “division of labor” among Notch components within the arthritic microenvironment. The consistent upregulation of Notch3 and Jagged1 expression is woven into the aggressive phenotype of RA-FLSs, whereas that of Jagged2 and Notch1 are primed to increase dynamically when alerted by local inflammatory triggers such as TNF-α or TLR4 signals or when cross-activated by ligand-rich neighboring cells such as macrophages.

**Fig. 1:**
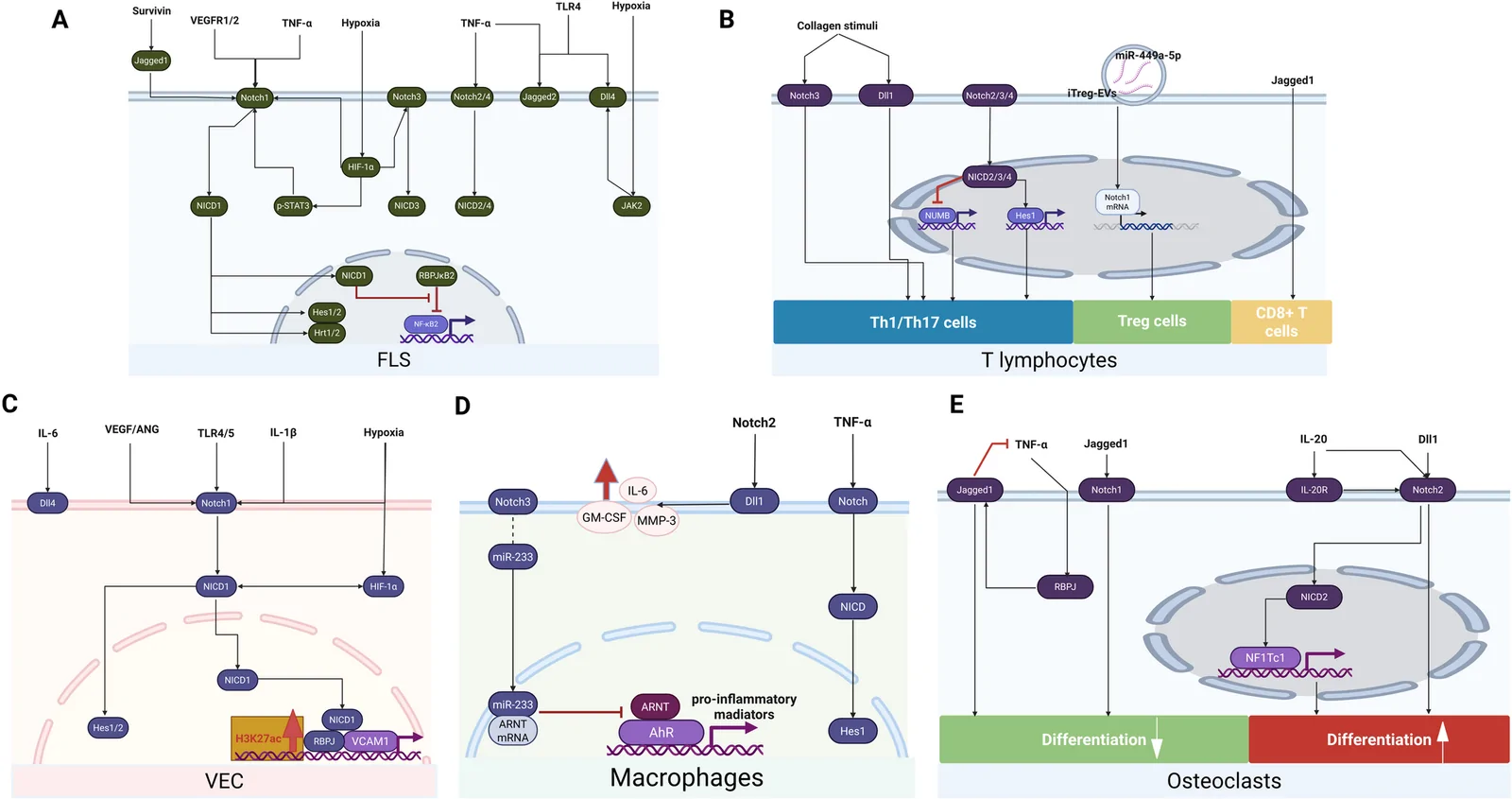
Schematic illustration detailing the specifics of the Notch signaling pathway in key cell types involved in the pathogenesis of RA: FLSs (**A**), T lymphocytes (**B**), VECs (**C**), macrophages (**D**), and osteoclasts (**E**)

On the basis of these expression patterns, researchers have further explored how different Notch components are involved in the “tumor-like” behaviors of RA-FLSs. Notch1 has been shown to be involved in regulating migration and epithelial‒mesenchymal transition in RA-FLSs, whereas Notch3 is involved in anti-apoptosis and pro-autophagy [8]. In RA-FLSs, TNF-α stimulates the nuclear translocation of NICD1 in a dose-dependent manner; however, interference via the γ-secretase inhibitor DAPT or antisense Notch1 oligodeoxynucleotide reduces IL-6 release and partially inhibits TNF-α-induced proliferation [13, 14]. The Notch1/RBPJ complex prevents inhibition of the NF-κB2 promoter, thereby activating FLS mitosis and proliferation [15]. Ando and colleagues reported that the TNF-α-induced activation of Notch1, Notch4, and Jagged2 may prompt FLSs to regain a primitive, invasive phenotype [16]. Survivin, a protein that is highly expressed in various tumors and exerts antiapoptotic effects, is considered a marker of highly proliferative, highly invasive tumor-like behaviors. Ma and colleagues reported that survivin is also highly expressed in RA synovial tissues and RA-FLSs and can promote RA-FLS proliferation and the expression of the angiogenic mediators VEGFR1, ANG1, and ANG2 through activation of the Notch1/Jagged1/Hes1 signaling pathway while inhibiting apoptosis in RA-FLSs [17]. Cross-talk between VEGFR1/2 and Notch1 is involved in the interaction between VECs and RA-FLSs mediated by syntetin-1—a newly discovered protein in RA joint synovial fluid—which induces inflammation [18]. Under conditions of inflammatory stress, TLR4 stimulation can further upregulate the expression of Notch ligands and receptors [12]. The mechanisms by which Notch signaling regulates FLS behavior in RA are illustrated in Fig. 1A.

#### Notch signaling pathway and VECs

Notch signaling in endothelial cells critically contributes to VEC activation, facilitating pathological angiogenesis and inflammatory infiltration, which are essential for RA progression.

Pathological evidence from the human RA synovium indicates the abundant expression of Notch1, Dll4, and Hes1, which are particularly localized within VECs around synovial blood vessels [6]. Further clinical profiling revealed that all the Notch receptors and ligands are present on VECs, establishing a structural basis for the potential involvement of these cells in synovial angiogenesis [12]. The functional implications of these patient-derived findings have been extensively explored through targeted experimental interventions. These studies have demonstrated that Notch1 mediates VEC invasion and formation into blood vessels induced by VEGF/ANG2 in patients with RA [19]. Furthermore, Notch signaling appears to be dynamically regulated in VECs. TLR4/5 binding can restructure the expression of Notch components, while the proinflammatory protein IL-6 has been shown to upregulate Dll4 transcription [12, 20]. In vitro networks of VEGFR1/2 and Notch1 are responsible for the cross-talk between syntenin1-rewired endothelial cells and RA-FLSs, promoting inflammation and angiogenesis [18]. The Notch1-RBPJ complex binds synergistically with the NF-κB subunit RELA to promote the acetylation of the histone H3K27, thereby exacerbating adhesion molecule expression in VECs and perivascular pathogenic leukocyte infiltration [21]. These findings confirm that Notch1-mediated chromatin remodeling is a key epigenetic mechanism that promotes chronic inflammation. The mechanisms through which Notch signaling regulates VEC behaviors in RA are shown in Fig. 1C.

### The Notch signaling pathway and immune cells

The Notch signaling pathway serves as a central orchestrator of immune cell fates, shifting homeostatic balance between pro- and anti-inflammatory axes during RA progression.

#### The Notch signaling pathway and T lymphocytes

The Notch signaling pathway regulates the proliferation, differentiation, activation, and functioning of T lymphocytes through direct gene induction or cross-talk with other pathways (Fig. 1B). The effects of Notch signaling on Th17/Treg balance and autoreactivity are critically shaped by specific pathway components and target cell types. Clinical characterization of T-cell subsets in RA patients has revealed a distinct signature of Notch dysregulation: specifically, the expression and activation of Notch2/3/4 are increased in the Th cells of individuals with active RA, with activated T lymphocytes serving as the primary source of increased Notch3 expression [22]. Furthermore, compared with healthy controls, RA patients exhibit significantly higher expression of Hes1 in Th cells and lower expression of the negative regulatory gene NUMB, regardless of disease activity [22]. To elucidate the causal mechanisms that underlie these clinical trends, in vivo studies have demonstrated how precise interventions that target the Notch pathway can selectively rebalance pathogenic and regulatory T-cell responses. Delivery of TGF-β-induced iTreg extracellular vesicles (iTreg-EVs) enriched with miR-449a-5p to arthritic mice restored the Th17/Treg balance by directly silencing Notch1 mRNA in recipient T cells [23]. This role of the Notch pathway is further underscored by the fact that both genetic knockout of Notch1 and pharmacological inhibition of γ-secretase alleviates arthritis in CIA and CAIA models, a benefit directly tied to marked Treg expansion [24]. Jiao and colleagues reported that DAPT treatment significantly inhibited the inflammatory response of Th1 and Th17 cells in the spleen and lymph nodes of CIA mice, whereas treatment with Dll3 significantly increased the expansion frequency and absolute number of Th17 cells among naive T cells [25]. Subsequent studies further demonstrated the involvement of Notch3 and Dll1 in the expansion of Th1 and Th17 cells induced by type II collagen-specific restimulation [26]. Crucially, the role of the Notch pathway is not unidirectionally proinflammatory; supplementation with Jagged1 delays CIA progression by modulating CD8 + T-cell responses, whereas its blockade exacerbates the disease [27]. These contrasting outcomes underscore that the pathogenic versus protective role of Notch signaling is highly ligand-dependent and cell-type specific. While activation of the Dll/Notch3 axis primarily drives CD4 + Th1/Th17 expansion, Jagged1 exerts a protective effect by transmitting an indirect negative signal to CD8 + T cells without altering CD4 + T-cell responses.

#### The Notch signaling pathway and macrophages

Within the microenvironment of arthritic joints, the phenotypic polarization and proinflammatory secretion of macrophages are tightly regulated by localized Notch ligand–receptor interactions. Clinical evaluations of patients with RA have revealed a link between specific macrophage-associated Notch signaling signatures to disease pathology. Zack and colleagues reported that the expression of Notch ligands on macrophages isolated from the RA synovium could serve as a potential biomarker for anti-TNF-α therapy, likely reflecting activation of Notch receptors on neighboring RA-FLSs and VECs [12].

Mechanistic studies involving patient-derived cells and arthritis models have explained how Notch signaling influences macrophage behavior and related joint destruction. Downregulation of miR-223 in RA macrophages is associated with increased Notch signaling activity, where miR-223 can target and bind to the mRNA of aryl hydrocarbon receptor nuclear translocator (ARNT), inhibiting the expression of proinflammatory mediators that themselves are mediated by the AHR/ARNT pathway in macrophages [28]. In CIA mice, the interaction between Dll1 in joint-localized macrophages and Notch2 in FLSs promotes the secretion of IL-6, GM-CSF, and MMP-3; consequently, Dll1 blockade can specifically inhibit these mediators and slow disease progression [29]. Sun and colleagues further demonstrated that Notch signaling activation occurs predominantly in proinflammatory M1-type macrophages and that inhibiting the Notch pathway successfully mitigates TNF-α-induced polarization and joint bone destruction in Hes1-GFP/TNF-Tg mice [30]. The mechanisms through which Notch signaling regulates macrophage behaviors in RA are shown in Fig. 1D.

### The Notch signaling pathway and osteoclasts

Pathological bone erosion in RA is regulated by Notch signaling, which exerts divergent control over osteoclasts and osteoblasts depending on the receptor–ligand interactions that occur. Recent investigations highlight a nuanced dual role of Notch in pathological bone erosion. Experiments involving mesenchymal stem cells derived from RA patients have revealed that chronic Notch activation is closely linked to impaired osteoblast differentiation and heightened inflammatory bone loss [31], while the administration of Notch inhibitors prevented bone loss and osteoblast inhibition in TNF-α transgenic mice [31]. Furthermore, IL-20 promotes osteoclast differentiation through upregulation of the activity of the Notch2/NFATc1 pathway in a dose-dependent manner, accelerating bone erosion in patients with RA [32]. Several studies have shown that Notch signaling exerts protective effects that specifically inhibit osteoclastogenesis; for example, Ng and colleagues reported that recombinant Jagged1 inhibits inflammatory osteoclastogenesis [33], while Sekine and colleagues reported that Notch2/Dll1 activation promotes osteoclast formation, whereas enhanced Notch1/Jagged1 signaling inhibits it [34]. A mechanistic study revealed that Jagged1 can selectively bind to RBPJ, thereby linking the TNF and Notch pathways to inhibit TNF-mediated osteoclastogenesis via a feedback loop [33]. Although the protective efficacy of DAPT suggests that the proosteoclastogenic forces of the Notch2/Dll1 axis may predominate in inflamed joints [34], the findings described above show that Notch signaling regulates RA bone erosion through a delicate equilibrium between receptor–ligand interactions. The mechanisms through which Notch signaling regulates osteoclast behaviors in patients with RA are shown in Fig. 1E.

### Regulation of the Notch signaling pathway inhibits RA progression

Targeting the Notch signaling pathway to modulate immune and stromal cell functions represents a promising therapeutic strategy for mitigating progression in RA, as demonstrated by numerous preclinical intervention studies. Extensive research involving animal models has demonstrated the potential of targeting Notch signaling for alleviating arthritic symptoms. Ahmad and colleagues reported that the CXCR3-specific antagonist NBI-74,330 alleviates CIA in mice by reducing the proportions of CD4 + Notch3+ and CXCR3 + Notch3+ T lymphocytes in the spleen [35]. Systematic Notch inhibition with LY411575 or DAPT in CIA rats and mice has been shown to reduce joint structural damage and vascular leakage through suppression of the Notch1/3 and NF-κB pathways [36, 37]. Natural compounds such as nimbolide, triptolide, and norisoboldine, along with herbal formulas such as Fangjihuangqi Decoction and the Tibetan medicine QSD, have been shown to effectively suppress synovial inflammation and angiogenesis by targeting the Notch1 or Notch2 pathways [38–42]. The use of precision delivery systems have further improved these outcomes. For example, hyaluronic acid nanoparticles that carry DAPT or targeted micelles that harbor Notch1 siRNA significantly reduce systemic cytokine levels and arthritis scores in CIA models [43–45]. Recent developments include the use of ROS-sensitive polymeric micelles carrying triptolide, which inhibit proinflammatory M1 macrophage differentiation via the NF-κB/Notch1 axis and successfully mitigate cartilage degradation in vivo [46].

Despite these promising results in experimental arthritis models, the translation of these therapies into clinical practice remains substantially challenging. Clinical evidence regarding small-molecule Notch inhibitors, including OMP-52M51, OMP-59R5, and PF-06650808, is derived primarily from oncology trials, where treatment is frequently terminated because of severe side effects or insufficient therapeutic efficacy [20]. These safety concerns in humans have thus far limited the exploration of Notch-blocking strategies in clinical applications for managing RA. Consequently, a substantial gap remains between the high efficacy observed in preclinical rodent models and successful clinical translation into human RA therapies.

In summary, the Notch pathway plays a pivotal role in RA by modulating the cross-talk between immune cells and synovial stromal cells. Safely and precisely targeting this pathway is a promising therapeutic approach for suppressing chronic joint inflammation and progressive bone erosion.

## The Notch signaling pathway and systemic lupus erythematosus (SLE)

**Fig. 2:**
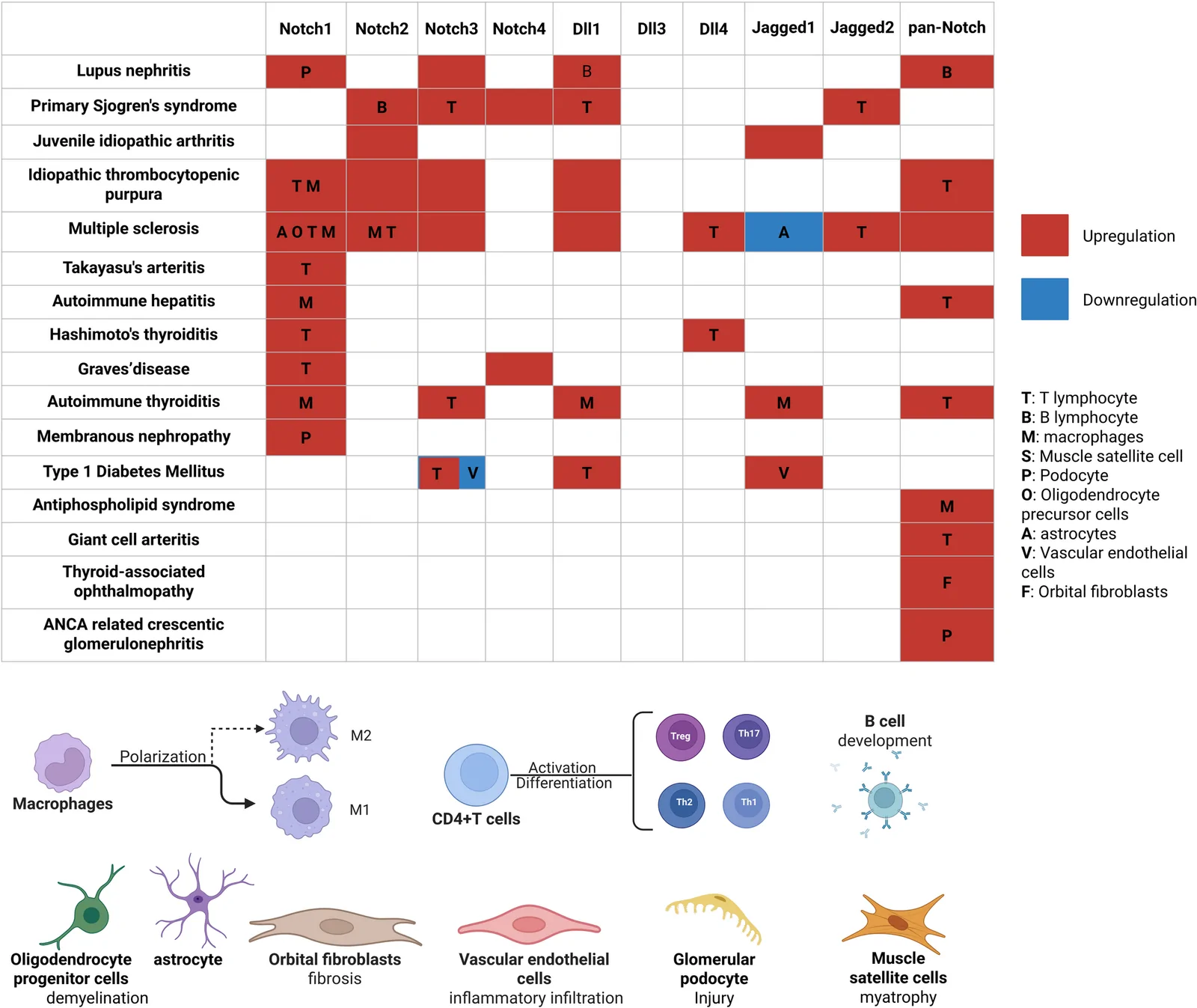
Expression features and pathogenic mechanisms of action of Notch signaling molecules across diverse autoimmune diseases. T (T lymphocytes): Associated with aberrant activation and lineage differentiation toward inflammatory subsets or impairment of regulatory T cells. B (B lymphocytes): Linked to abnormal B-cell development, activation, and autoantibody production. V (vascular endothelial cells): Responsible for triggering inflammatory-cell infiltration. P (glomerular podocytes): Mediate podocyte injury. O (oligodencrocyte precursor cells) and A (astrocytes): Implicated in demyelination. F (orbital fibroblasts): Drive fibrosis. S (muscle satellite cells): Involved in impaired muscle regeneration and myatrophy

Systemic lupus erythematosus (SLE) is a systemic autoimmune disorder characterized by the deposition of immune complexes that affect multiple organs, including the skin and kidneys as well as the central nervous system. Patients typically experience cellular and humoral immune dysfunction, resulting in the production of autoantibodies such as antinuclear and anti-double-stranded DNA and anti-Sm antibodies. In SLE, the Notch signaling pathway is involved in modulating systemic autoimmunity and end-organ damage, with its components playing distinct receptor-specific and context-dependent roles. For example, Notch1 is overactivated and colocalizes with podocytes in the glomeruli of patients with clinical lupus nephritis (LN) [47]; Notch3 is similarly overexpressed in LN glomeruli [48]. Experiments in lupus mouse models have demonstrated that targeting different components of this pathway can alter LN progression (Table 2). In BALB/C-lpr mice, inhibition of Notch1 expression has been shown to increase corticosteroid sensitivity and alleviate the symptoms of LN during glucocorticoid treatment [49]. Wu and colleagues revealed that inhibiting Notch1 expression in MRL/lpr mice reduces renal podocyte injury by suppressing NLRP3 inflammasome activation [47]. The injury induced by IgG in the podocyte cell line MPC5 can be mitigated via miR-30a through the downregulation of Notch1 expression [50]. However, in contrast to the result of Notch1 deficiency, systemic Notch3 deficiency in B6.Fas^*lpr/lpr*^ mice exhibit more severe lupus manifestations and increased mortality [48]. This phenotypic discrepancy can be attributed to the protective feedback loop of the Y-box binding protein-1 (YB-1)/Notch3 axis, wherein guanidinylated YB-1 in individuals with active SLE specifically activates Notch3 signaling in T cells to promote the expression of anti-inflammatory mediators and maintain T-cell homeostasis by expanding regulatory T cells while limiting the number of pathogenic double-negative T cells [48]. Experimental data indicate that ILC3s originating from the intestine aggregate in the kidneys during LN, where they facilitate B-cell differentiation and autoantibody production through a Dll1/Notch-dependent mechanism [51]. In conclusion, the selective targeting of specific Notch receptors or ligands is a potential therapeutic strategy for correcting immune cell dysregulation, reducing autoantibody production, and mitigating localized tissue injury in SLE (Fig. 2).

Antiphospholipid syndrome (APS), which is frequently associated with SLE, is a systemic condition characterized by elevated antiphospholipid antibodies and a high risk of thrombosis. In APS, Notch signaling is involved in sEV-induced monocyte activation and systemic inflammation (Fig. 2; Tables 1 and 2). Clinical profiling of patients with primary APS has revealed an increase in circulating platelet-derived small extracellular vesicles (sEVs), which exhibit significantly low levels of miR-326 [52]. Mechanistically, the loss of vesicle-encapsulated miR-326 activates the Notch pathway in recipient monocytes, facilitating the production of inflammatory mediators such as NFKB1, MCP1, and IL-12 [52]. However, this evidence reveals only an indirect association between the Notch pathway and APS, and the precise expression status of the components of this pathway in APS patients remains to be validated through clinical observational studies.

## The Notch signaling pathway and primary Sjögren’s syndrome (pSS)

Primary Sjögren’s syndrome (pSS) is a chronic inflammatory autoimmune disorder characterized by abnormal lymphocyte proliferation and progressive damage to exocrine glands, primarily the salivary and lacrimal glands. Abnormal Notch signaling acts as a critical orchestrator of various processes in pSS by regulating the proliferation, trafficking, and function of downstream B- and T-cell subsets, promoting exocrine gland destruction and systemic inflammation. Clinical investigations involving patients have provided substantial evidence of the involvement of the Notch pathway in pSS (Table 1). Luo and colleagues performed a genome-wide DNA methylation analysis of PBMCs from pSS patients and reported significant differences in the methylation of genes enriched in Notch signaling, especially among individuals with high serum IgG levels [53]. Genome-wide association studies in the Chinese Han population revealed that the Notch4 gene is associated with pSS [54]. Histological analysis of salivary gland and tonsil samples from pSS patients revealed that Notch2 mRNA is expressed in the tonsils and that its expression is correlated with the abundance of transitional type II B cells in the salivary glands [55]. Mechanistic studies involving animal models have further clarified how the components of the Notch pathway influence pSS progression (Table 2). Whole-gene transcriptome analysis of lacrimal glands in C57BL/6.NOD-Aec1Aec2 mice showed that a temporary increase in the activity of the Notch2/NF-κβ14 pathway in marginal zone B cells (MZBs) during early stages is essential for MZB migration to the lacrimal glands [56]. The presence of Notch2-positive cells in the salivary glands in this model coincides with the formation of lymphocytic foci, which subsequently facilitates autoimmune T-cell responses against exocrine tissues [57]. Furthermore, Wang and colleagues demonstrated that decreased pulmonary function is accompanied by a reduced proportion of peripheral Tregs and lower numbers of CD4 + CD25+Notch1 + and CD4 + CD25+Notch3 + T lymphocytes in pSS rat models. This systemic reduction was associated with significant increases in the expression of Notch3, Dll1, and Jagged2 in lung tissues [58]. These findings show that the Notch pathway plays a key role in promoting immune cell migration and systemic autoimmunity in pSS (Fig. 2). Targeting this pathway is a promising strategy for preventing exocrine gland destruction and treating severe extraglandular complications.

## The Notch signaling pathway and juvenile idiopathic arthritis (JIA)

Juvenile idiopathic arthritis (JIA) is a common pediatric autoimmune condition characterized by chronic polyarthritis and systemic involvement, including fever, rash, lymphadenopathy, and splenomegaly. Dysregulated Notch signaling is tightly associated with lymphocyte imbalances that promote systemic and joint inflammation in children with JIA (Fig. 2). The mRNA expression of Notch2, Jagged1, and Hes1 in PBMCs from patients with JIA is significantly elevated compared with that in PBMCs from healthy children, which coincides with an increase in the plasma levels of cytokines such as IL-1β, IL-10, IL-6, IL-17, and TGF-β [59]. In patients with systemic JIA, Hes1 mRNA expression positively correlated with STAT3 mRNA expression as well as with the numbers of CD3 + T lymphocytes, CD19 + B lymphocytes, CD3 + CD8+ Tregs, and CD16 + CD56+ NK cells [59]. Further analyses of peripheral blood from individuals with oligoarticular JIA revealed that Hes1 mRNA expression is also positively correlated with the number of CD3 + T lymphocytes [59]. These clinical findings underscore the likely role of the Notch signaling pathway in lymphocyte subpopulation imbalance and the dysregulation of the secretion of related cytokines in JIA (Table 1). While these patient-derived associations offer valuable insights into the nature of the disease environment, further mechanistic investigations in experimental models would be beneficial for precisely determining the potential causal relationships between Notch signaling and inflammatory phenotypes. Targeting this aberrant Notch activation offers a promising therapeutic avenue for restoring immune homeostasis in JIA patients.

## The Notch signaling pathway and idiopathic thrombocytopenic purpura (ITP)

Idiopathic thrombocytopenic purpura (ITP) is an acquired autoimmune disease characterized by the excessive destruction of platelets that have become sensitized to autoantibodies by the mononuclear phagocyte system. Clinical manifestations primarily include thrombocytopenia, which is frequently accompanied by hemorrhage in the skin and mucosal membranes. The precise regulation of T-cell fate is essential for restoring immune tolerance in ITP, a process in which the Notch signaling pathway plays a critical regulatory role (Fig. 2; Table 1). Clinical data have revealed the elevated expression of Notch1 and Hes1 in both pediatric and adult ITP, with the former also exhibiting a strong positive correlation in the expression levels of these markers during initial clinical visits [60, 61]. Another clinical study indicated that even after effective treatment, the abnormal upregulation of Notch1 expression may not return to normal [62]. This difference between clinical remission and molecular persistence suggests that current standard therapies, while effective at transiently restoring platelet counts, may not adequately address the underlying Notch-related immune dysregulation. Intriguingly, the pathogenic increase in the activity of Notch signaling is not universal across all T-cell subsets; among lymphocytes, for example, researchers have reported a significant decrease in peripheral CD4 + CD25+CD127low Treg counts in ITP patients, which was associated with reduced Notch1 transcription [63], suggesting a potential protective role of Notch1 signaling in maintaining Treg homeostasis. Genome-wide DNA methylation profiling of CD4 + T lymphocytes revealed epigenetic alterations in genes enriched in the Notch pathway [64]. High-throughput methylation analysis revealed that compared with that in control individuals, the Notch1 gene in individuals with IPT significantly differed in methylation status [65]. Wang and colleagues reported that the level of Notch1/2/3 mRNA in the PBMCs of patients with acute ITP was significantly greater than that in the PBMCs of healthy controls and of patients with chronic ITP [66]. In addition to these observational markers, experimental studies have confirmed that Notch signaling acts as an important modulator of T-cell polarization (Table 2). The results of a mechanistic study suggested that the pathogenesis of ITP may alter Th1/Th2 differentiation through methylation changes in Notch1 regulatory regions [65]. The Notch signaling pathway participates in the pathogenesis of acute ITP by regulating Th22 differentiation through the aryl hydrocarbon receptor (AhR) [66]. Furthermore, DAPT can suppress the differentiation of CD4 + T cells into Th17 cells [67], while the lncRNA PVT1 can bind to Notch1 and accelerate its degradation to inhibit Th17 polarization [68]. The clinical translation of these findings requires further confirmation, however, and whether the postremission persistence of Notch alterations reflects a core pathogenic driver or a secondary molecular feature remains to be fully elucidated through further studies. Nevertheless, targeting the Notch pathway is a prospective strategy for correcting T-cell imbalances and restoring immune tolerance in individuals with ITP.

## The Notch signaling pathway and multiple sclerosis (MS)

Multiple sclerosis (MS) is a chronic autoimmune disorder characterized by central nervous system inflammation, demyelination, and axonal damage. In MS, aberrant Notch signaling is associated with both neuroinflammation and remyelination failure (Tables 1 and 2). Clinical evidence has revealed a significant association between the production of nADA and Notch2 expression in CD14 + monocytes, which coincides with an increased proportion of proinflammatory monocyte subsets across multiple MS cohorts [69]. Extensive research involving experimental autoimmune encephalomyelitis (EAE) models has targeted various Notch pathway components. For example, the suppression of CCR5 expression reduces activity in the NF-κB/Notch signaling pathway in the brain and spleen, thereby improving central nervous system inflammation in EAE mice [70]. The targeted blocking of Dll4 with monoclonal antibodies has been shown to expand Treg subsets, promoting Th2 and Treg immune responses while impeding Th1- or Th17-mediated damage in EAE mice [71]. IL-17R interacts with Notch1 through its extracellular domain to promote IL-17R/Act1/NICD1 complex formation. Upon translocation to the nucleus, this complex recruits the STEAP4 promoter to promote inflammatory cell proliferation [72]. Epigenetic and transcriptomic studies have further revealed that the lncRNA Gm13568, whose expression is upregulated in EAE mice, regulates cytokine production in activated astrocytes via the Notch1/STAT3 pathway to promote demyelination [73]. Research on neural repair and myelination has suggested that the nonclassical Notch ligand contactin on the membranes of demyelinated axons activates Notch1 on oligodendrocyte progenitor cells (OPCs); however, the nuclear translocation of NICD1 can be inhibited by the proapoptotic factor TIP30, subsequently stalling differentiation and remyelination [74]. Notch signaling also coordinates with the mTORC1 pathway to orchestrate the fates of OPCs. This cross-talk integrates environmental and nutrient cues to determine whether OPCs maintain their progenitor state or differentiate into myelinating oligodendrocytes. Specifically, activation of the mTORC1 pathway promotes expression of the Notch ligand Dlls, thereby modulating Notch-mediated cell fates in response to metabolic status [75]. Furthermore, extrinsic factors such as bone morphogenetic protein 4 (BMP4) inhibit remyelination by promoting histone acetylation and reducing histone deacetylase recruitment at the promoter regions of Notch target genes. This epigenetic activation sustains Notch signaling, which subsequently guides the fates of OPCs toward astrogliogenesis rather than oligodendrocyte maturation in demyelinated environments [76]. Similarly, downregulation of Notch1 mRNA expression in acute demyelinating models reduces Hes1 and Jagged1 expression while increasing the expression of the oligodendrocyte marker Sox10, indicating that the suppression of Notch1 expression promotes remyelination [77]. Finally, genetic interventions, such as the induction of MyeNotch1KO in mice or Notch1 deficiency in macrophages, have been shown to reduce the extent of spinal cord pathology and Th1/Th17 infiltration, effectively restricting the progression of EAE [78, 79].

Different Notch ligands appear to exert opposing immune regulatory effects. Dll1 predominantly mediates pathogenic Th1 immune responses, whereas Jagged1 exerts peripheral and central protective effects. The antibody blockade of Jagged1 exacerbates central nervous system (CNS) inflammation, whereas administration of exogenous Jagged1-Fc fusion protein significantly delays EAE onset and alleviates the clinical severity of the condition, which is closely linked to the specific induction of anti-inflammatory Tr1 and Th2 cell expansion [80]. Furthermore, CNS-resident astrocytes specifically express high levels of Jagged1, which can be markedly increased further by the anti-inflammatory cytokine TGF-β; however, during the course of EAE, astrocytic Jagged1 expression is downregulated via feed-forward inhibition by proinflammatory cytokines, leading to the collapse of central, intrinsic protective mechanisms [80]. Conversely, Dll1 aggravates the disease by increasing the proportion of Th1 cells in the CNS; thus, anti-Dll1 antibodies exhibit protective effects in EAE mouse models [80]. Another study demonstrated that Jagged2-activated NICD1 interacts with Smad3 to transactivate the Il-9 promoter, thereby driving Th9 cell differentiation [81]. Notably, the ultimate pathological outcome of this cooperative Notch/Smad3 signaling in EAE is strongly dictated by the spatiotemporal context of the microenvironment; for example, pretreatment with an agonistic anti-Jagged2 antibody prior to immunization significantly suppresses EAE progression by increasing the number of Tregs and the Treg/Th17 ratio [81], whereas concurrent activation of the Jagged2 pathway during immunization facilitates pathogenic Th17 cell generation. In this inflammatory milieu, TGF-β cooperates with high levels of IL-6, thereby exacerbating the clinical severity of EAE in an IL-9-dependent manner [81]. This dual regulation, involving both timing and the cytokine microenvironment, further demonstrates the precise, bidirectional modulatory role of the Notch pathway in EAE [81]. Since different Notch components have distinct effects on immune injury or repair (Fig. 2), successful therapeutic translation will require the precise targeting of targeting specific ligands or receptors rather than the implementation of pan-Notch inhibition.

## The Notch signaling pathway and autoimmune vasculitis

The pathological characteristics of vasculitis include inflammatory cell infiltration around the vascular wall as well as vascular damage, which manifests as fibrin deposition, collagen fibrosis, and necrosis of endothelial and smooth muscle cells. The Notch pathway not only promotes vascular inflammation by regulating the metabolic reprogramming and pathogenic differentiation of infiltrating T lymphocytes in vasculitis but also actively regulates the survival and functional integrity of resident cells in various organs secondary to microvascular injury.

Clinical evidence from histological biopsies has revealed that the Notch receptors Jagged1 and Dll1 are highly expressed in the temporal arteries of individuals with giant cell arteritis (GCA), where abundant NICDs are present within infiltrating T lymphocytes [82]. In patients with Takayasu’s arteritis (TAK), CD4 + T cells tend to spontaneously differentiate into Th1 or Th17 cells with an extended lifespan [83]. Research on these clinical samples has revealed that excessive activation of the Notch1/AMPK pathway in T lymphocytes enhances mitochondrial metabolism and increases their survival capability, a potential mechanism that underlies the abovementioned spontaneous differentiation [83]. The results of in vivo experiments have also emphasized the critical role of the Notch pathway in autoimmune vasculitis (Table 2). In GCA murine models, disruption of the Notch pathway through inhibition of intracellular domain cleavage or the use of Jagged1-Fc to compete with receptor–ligand interactions significantly decreases Th17 and Th1 immune responses [82]. Notch1 signaling has also been identified as a primary regulator of CD4 + T-cell-intrinsic mTORC1 hyperactivity in TAK [84]. Notch1 not only promotes the expression of mTOR but also facilitates its lysosomal localization, thereby fueling the spontaneous differentiation of pathogenic Th1 and Th17 cells. Therapeutic blockade of the Notch-1/mTORC1 axis, through either γ-secretase inhibitors or genetic knockdown, has been shown to effectively attenuate vascular inflammation in humanized TAK chimeras [84].

When vasculitis affects the small vessels in the kidney, it manifests as renal inflammation and damage. Within the context of human antineutrophil cytoplasmic antibody (ANCA)-related crescentic glomerulonephritis, clinical studies have identified the urinary biomarker Gremlin-1 as a significant indicator of disease progression [85]. To this end, Notch signaling acts as a regulator of renal cell survival, modulating podocyte resistance to glomerular injury (Fig. 2). Research involving experimental models has described the regulatory roles of the Notch pathway under these conditions (Table 2); in mice with anti-glomerular basement membrane nephritis induced by the administration of nephrotoxic serum, the bromodomain and extraterminal domain inhibitor JQ1 effectively mitigated the overexpression of Gremlin-1. This intervention was associated with a reduction in glomerular damage and restoration of the number of renal podocytes [85]. The results of mechanistic analysis revealed that JQ1 achieves these effects by inhibiting the expression of Hes1 and Hey1 in the injured kidney [85]. While Notch activation contributes to the progression of ANCA-associated crescentic glomerulonephritis, the role of Notch signaling in podocyte injury appears to be environment dependent. This differs from membranous nephropathy (MN), a disease that is primarily driven by autoantibodies that target the glomerular basement membrane, wherein Notch1 expression is reportedly downregulated in the renal tissues of model rats [86]. This discrepancy suggests that Notch-based regulation is complex and may affect podocyte survival differently depending on the specific autoimmune microenvironment. Current evidence, however, was predominantly obtained through indirect interventional results from the non-Notch-specific inhibitor JQ1 in animal models; given the lack of direct causal evidence from patients, the differential regulation of the Notch pathway in podocyte injury remains at the level of phenotypic description.

## The Notch signaling pathway and autoimmune hepatitis (AIH)

AIH is a chronic progressive inflammatory liver disease characterized by increased serum transaminases, hypergammaglobulinemia, and the presence of autoantibodies. Histological examinations typically reveal interface hepatitis with lymphocytic and plasma cell infiltration, which can rapidly lead to cirrhosis and liver failure in severe clinical cases. Although direct genetic or pharmacological evidence that Notch triggers AIH is lacking, the results of several experiments have provided indirect evidence of this relationship. In AIH mice, for example, miR-20b has been shown to bind the 3’-UTR of Notch1 mRNA to inhibit its expression, thereby suppressing macrophage activation and tissue infiltration to mitigate inflammation and fibrosis [87]. Posttranslational modifications have also been shown to play a significant role in protein homeostasis. Rats deficient in O-GlcNAc glycosylation exhibit increased susceptibility to concanavalin-A induction, resulting in liver injury and delayed repair patterns resembling those in human disease. The results of experiments with these models, in addition to those of in vitro experiments, suggest that increased CD4 + T-cell infiltration and impaired Treg activation are linked to the inhibition of Notch signaling within CD4 + T cells [88]. These observations imply that Notch signaling may influence AIH-related immune responses in two ways, promoting proinflammatory macrophage activity while preserving Treg-mediated regulation (Fig. 2; Tables 1 and 2). Further mechanistic analyses are warranted before these results can be translated into a potential therapeutic direction for AIH to improve precision intervention and immunomodulation.

## The Notch signaling pathway and autoimmune thyroid disease (AITD)

Autoimmune thyroid disease (AITD) encompasses a group of autoimmune conditions, including Hashimoto’s thyroiditis, Graves’ disease (GD), thyroid-associated ophthalmopathy (TAO), and autoimmune thyroiditis. These disorders are characterized by a form of immune dysregulation that results in the production of autoantibodies that target thyroid antigens, such as thyroglobulin antibodies (TgAb) and thyroid microsomal antibodies. The results of clinical genetic studies have revealed a significant relationship between the SNP rs424232 of Notch4 and young-age-onset Graves’ disease [89]. In patients with Hashimoto’s thyroiditis, the mRNA levels of Notch1, Dll4, and ROR-γt in PBMCs are markedly greater than those in healthy individuals. The expression of these markers is positively correlated with the proportion of Th17 cells and the serum titers of both thyroid peroxidase antibodies and TgAb [90]. The elevated expression of miR-363 in the peripheral blood Tregs of patients with GD indicates that this signaling axis is closely linked to immune tolerance abnormalities in human disease [91]. The results of an in vitro revealed that miR-363 regulates the proliferation and differentiation of Tregs by binding to STAT4 mRNA, modulating the STAT4/hspb1/Notch1 axis [91]. Notch signaling affects the metabolic and functional programming of Th17 cells, as its activation leads to the hyperphosphorylation of AKT and mTORC1 effectors, thereby fueling Th17-mediated thyroid damage [92]. Insights from experimental autoimmune thyroiditis (EAT) models indicate that transplanting OX40L+Jagged1 + bone marrow-derived cells can promote Treg expansion via OX40 and Notch3 signaling, effectively delaying disease onset [93]. Conversely, treatment with the inhibitor DAPT reduces the severity of thyroiditis and the proportion of Th17 cells among the splenic mononuclear cells of EAT mice, where the latter Th17 is correlated with serum TgAb and Notch1 expression [94]. This apparent discrepancy may stem from the functional divergence of Notch receptor paralogs across different cell lineages; specifically, while Notch3 activation preferentially sustains Treg-mediated immune tolerance, Notch1 signaling predominantly drives pathogenic Th17 cell differentiation. Therefore, the therapeutic efficacy of pan-Notch blockade by DAPT likely reflects the major influence of the proinflammatory Notch1/Th17 axis during autoimmune injury. Additionally, excess iodine accelerates thyroiditis in NOD.H-2h4 mice by increasing Notch-mediated M1 macrophage polarization [95]. Studies on primary orbital fibroblasts from late-stage TAO have demonstrated that the antifibrotic drug serelaxin can counteract inflammatory responses and fibrosis by suppressing activation of the Notch pathway [96]. Notch signaling plays a dual and fine-tuned role in the pathogenesis of AITD pathogenesis by modulating the Th17/Treg balance and macrophage-mediated inflammation (Fig. 2; Tables 1 and 2). Regulating this signaling network is a promising strategy for arresting autoimmune-mediated thyroid destruction and associated fibrotic complications.

## The Notch signaling pathway and type 1 diabetes mellitus (T1DM)

Type 1 diabetes mellitus (T1DM) is an autoimmune condition characterized by insulin deficiency and progressive vasculopathy and serves as a central driver of numerous systemic complications. The Notch signaling network modulates this pathology through highly distinct downstream mechanisms (Tables 1 and 2). Clinical evaluations of muscle satellite cells in T1DM patients have revealed that a decrease in these progenitor cell populations coincides with a significant upregulation of the expression of Dll1, suggesting that aberrant Notch activation is linked to the muscle damage observed in humans with T1DM [97]. Insights from multiple experimental platforms have further characterized the underlying molecular shifts in vascular, immune and muscular tissues (Fig. 2). In T1DM mouse models, the downregulation of Jagged1 and Notch3 expression in vascular tissues has been shown to inhibit vessel arteriovenous specification and reduce vascular coverage [98]. In addition to vascular effects, the dysregulation of CD4 + CD25+ Tregs critically influences disease development. The results of in vivo investigations have indicated that activated Notch3 signaling positively regulates Treg differentiation through the pTα/pre-T-cell receptor pathway in mice, with CD4 + Notch3+ T lymphocytes demonstrating the ability to inhibit hyperglycemia and insulin resistance in laboratory settings [99]. Moreover, research using Akita mice has confirmed that Notch activation via Dll1 is intrinsically associated with diabetic muscle atrophy [97]. Although the results of these studies have provided a robust framework for understanding the role of Notch signaling in T1DM, the specific methodologies employed, such as the use of the Akita mouse model, focused predominantly on the metabolic stress that occurs in insulin deficiency rather than the chronic autoimmune destruction typical of the human pathology. These insights demonstrate that the functional outcomes of Notch signaling in T1DM are profoundly shaped by the tissue microenvironment, which is characterized by a distinct dual nature across different anatomical sites. Therapeutic interventions must selectively target these tissue niches for alleviating vascular and muscular complications in T1DM without disrupting systemic immune homeostasis.

## Cross-talk between the Notch and canonical regulatory pathways

Upstream epigenetic and autophagic regulation influences the expression levels of multiple Notch signaling components. The activated Notch target genes subsequently interact with the Wnt and NF-κB/NLRP3 signaling pathways to precisely modulate chronic inflammation. These signals eventually converge on the downstream PI3K/AKT/mTORC1 axis to regulate metabolic reprogramming and immune homeostasis. This section explores the intricate interplay between the Notch signaling pathway and the NF-κB, NLRP3 inflammasome, autophagy, and Wnt pathways, highlighting how these interactions promote or resolve chronic inflammation.

As an element of upstream regulatory mechanisms, epigenetic modifications and cellular degradation systems participate in modulating the activation of Notch signaling. Alterations in DNA methylation status can significantly influence the binding affinity of key transcription factors, such as RBPJ [100]. These modifications can lock the signaling pathway in a constitutively active state and ultimately influence immune cell fates and differentiation processes. In uveitis, the DNA demethylase TET2 induces hypomethylation within the Notch1 promoter, which leads to the overexpression of Notch1 mRNA [101]. This modification locks the signaling pathway in an active state, subsequently promoting the differentiation of naive CD4 + T cells into Th17 cells and thus skewing the Th17/Treg ratio [101]. In addition to epigenetic regulation, a sophisticated reciprocal relationship exists between Notch signaling and autophagic–lysosomal biogenesis. The coordinated lysosomal expression and regulation (CLEAR) network, modulated by the transcription factor TFEB, is essential for proper Notch localization and signaling. The TFEB-mediated regulation of V-ATPase subunits maintains the endolysosomal compartment; specifically, its depletion impairs ligand-independent Notch activity [102]. Conversely, Notch1 itself is a direct substrate for autophagic degradation via ATG16L1-positive vesicles. Defects in autophagy proteins, such as ATG7 or ATG16L1, lead to the pathological accumulation of NICD, driving the pathway hyperactivity observed in Crohn’s disease [103]. This protein buildup further primes the NLRP3 and NF-κB pathways, creating a feedback loop in which reduced autophagic flux fuels persistent immune activation and subsequent tissue fibrosis [104].

Notch signaling interacts with inflammatory and morphogenic pathways in autoimmune pathologies. Integration of the inflammasome results in the formation of a coordinated regulatory network essential for modulating the chronic inflammatory state. The NF-κB family, which is typically maintained in an inactive cytoplasmic state by IκB proteins, serves as a primary substrate for Notch-mediated modulation. This interaction is substantially facilitated by the coactivator Mastermind-like 1 (MAML1), which acts as a molecular bridge that increasing the transcriptional capacity of the p65 subunit and promotes IκBα degradation, thereby amplifying the responsiveness of NF-κB to inflammatory stimuli [105]. This Notch-driven NF-κB activation provides the necessary priming for the NLRP3 inflammasome, a pivotal sensor responsible for the maturation of IL-1β and IL-18. In conditions such as intestinal inflammation and experimental autoimmune uveitis, Notch activity serves as a critical modulator that connects these two systems into a consolidated Notch/NF-κB/NLRP3 axis [106, 107]. Within this axis, Notch signaling coordinates macrophage polarization and innate immune responses; specifically, its inhibition has been shown to concurrently suppress NF-κB-dependent transcription and NLRP3-mediated pyroptosis. Furthermore, Notch1 signaling balances this proinflammatory cascade with vascular restoration, where its attenuation suppresses the NLRP3/caspase-1 axis while promoting VEGF-mediated angiogenesis [108]. In addition to these inflammatory mediators, activation of the Notch signaling cascade is further complicated by its synergy with other morphogenic signals pathways that regulate tissue remodeling. Cross-talk between the Notch and Wnt/β-catenin signaling pathways is characterized by the direct engagement of β-catenin with the NICD and CSL, which influences cell fate [109]. In systemic sclerosis, this is evidenced by the BACE1/APP axis, wherein cleavage of the Wnt decoy receptor APP stabilizes β-catenin, subsequently triggering the expression of the Notch ligand Jagged1 for accelerating myofibroblast differentiation [110].

This regulatory network further extends to cellular bioenergetics, where the Notch signaling pathway intersects with the PI3K/AKT/mTORC1 axis to modulate immunometabolism. In AITD, Notch-induced Hes1 expression suppresses PTEN, leading to AKT/mTORC1 activation and pathogenic Th17 differentiation [92]. In TAK, Notch1 expression increases mTORC1 activity and its lysosomal localization to fuel Th1/Th17-mediated vascular inflammation [84]. This metabolic synergy further extends to neuroautoimmunity; in multiple sclerosis, mTORC1 integrates nutrient cues to promote Dlls expression, thereby modulating the Notch-dependent fate and remyelination of OPCs [75]. Understanding these interconnected signaling nodes is essential for developing comprehensive therapeutic strategies, as targeting individual pathways alone may be insufficient to control the complex pathogeneses of autoimmune diseases.

## Therapeutics and translation targeting the Notch pathway

The fundamental ability of the Notch signaling pathway to orchestrate cell fates primarily within immune populations, as well as within the stromal cells of target organs, underscores its fundamental role within the immune system, where it dynamically shapes both the strength and the quality of diverse immune responses. While therapeutic interventions that target this pathway have historically been developed in the context of oncology, the pathological role of Notch signaling in immune and immune-mediated inflammatory disorders is an established and expanding paradigm that can lead to unique therapeutic opportunities. From a translational perspective, the principles that underlie the targeting of Notch signaling in immune and inflammatory conditions operate under entirely different rules than those related to cancer treatment. In oncological therapies, achieving sufficient antitumor efficacy typically requires prolonged and systemic Notch inhibition, which requires an aggressive dosing schedule that frequently leads to the selection of resistant malignant cells through epigenetic variations and alternative survival mechanisms [111]. Conversely, the selection of resistant cellular clones is a negligible factor in the context of the immune system, thus allowing Notch blockade to be applied transiently or via intermittent bursts at sensitive, critical stages of immune cell differentiation and function [111]. This temporary intervention strategy provides the advantage of preserving vital lymphoid development and protective immune responses outside of the restricted treatment window while effectively reprogramming pathogenic immune cells into a less destructive state.

Despite this compelling therapeutic potential, the translation of these broad chemical interventions into successful clinical therapies is severely limited by profound structural and physiological limitations. The primary argument against the use of conventional pan-Notch inhibitors, such as broad-spectrum γ-secretase inhibitors, stems from their severe lack of substrate selectivity. The γ-secretase complex involves more than 149 distinct substrate candidates, including vital physiological molecules such as β-amyloid precursor protein, vascular endothelial growth factor receptor 1, ErbB4, CD44, PTK7, PDPN, and alcadein [112]. Because these alternative substrates are critical for human physiology, nonselective chemical inhibition will inevitably trigger severe, targeted toxicity-based mechanisms in healthy tissues [112]. Most notably, sustained systemic inhibition results in the profound accumulation of goblet cells within the small intestine, a condition that led to severe, dose-limiting diarrhea in patients enrolled in clinical trials. These profound toxicities have historically prevented the use of maximally effective therapeutic schedules, compromising overall treatment efficacy and resulting in the discontinuation of most completed and ongoing clinical trials. For example, trials evaluating prominent γ-secretase inhibitors, including BMS986115 and MK 0752, were terminated early during phase I because of these prohibitive dose-limiting effects [113]. Even candidates that have successfully advanced to phase II clinical trials, such as RO4929097, nirogacestat, and AL101, have ultimately yielded modest therapeutic efficacy when administered alone [113]. Furthermore, small-molecule interventions that target the downstream core components of this pathway, specifically the core Notch transcriptional activation complex, remain significantly limited in efficacy, while systemic blockade is frequently complicated by the compensatory activation of alternative signaling pathways [113].

To bypass the toxicity of systemic pan-Notch inhibition, experimental and preclinical studies have sought to pioneer highly precise, selective targeting strategies. To mitigate the side effects of nonselective inhibition, for example, researchers have developed humanized monoclonal antibodies that specifically target individual Notch1, Notch2, and Notch3 paralogs [113]. This highly specific targeting of individual receptors or ligands could allow a wider therapeutic reach than that achieved with systemic pan-Notch inhibition. For instance, the selective targeting of understudied pathway members, such as the Notch3 and Notch4 receptors, offers viable therapeutic opportunities even with prolonged inhibition, as blocking these specific receptors is not expected to induce severe adverse effects. Concurrently, allosteric regulation through γ-secretase modulators has been identified as a safer, highly selective therapeutic alternative. Unlike broad inhibitors, modulators do not suppress overall enzymatic proteolysis but instead fine-tune catalytic function to selectively alter cleavage specificity, achieving broad safety margins across multiple animal species [113]. Crucially, the implementation of tissue-specific prodrug strategies provides a powerful mechanism for bypassing systemic gastrointestinal toxicity. This approach is exemplified by Cpd164, a peptidomimetic prodrug designed as a substrate for aminopeptidase A and γ-glutamyl transpeptidase, peptidases that are highly expressed within damaged tissues. In preclinical models of acute kidney injury, Cpd164 undergoes rapid, localized transformation within the renal parenchyma rather than in the plasma, where it successfully downregulates the expression of Notch1 and cleaves the Notch intracellular domain to provide robust, organ-specific protection without inducing any overt side effects such as diarrhea [112]. In the future, the adaptation of innovative bioengineering frameworks originally developed for cancer immunotherapy will provide sophisticated blueprints for revolutionizing autoimmune care. These cutting-edge delivery paradigms include the administration of precise Notch modulators via customized nanoparticles to direct macrophage polarization or suppress harmful myeloid cell populations; the implementation of intermittent dosing regimens to prevent toxicity accumulation; the combination of Notch modulators and complementary immune checkpoint inhibitors to achieve synergistic regulatory effects; and the engineering of custom, synthetic Notch circuits into chimeric antigen receptor immune cells, including engineered T cells, natural killer cells, or macrophages, for achieving unprecedented cellular precision at the specific sites of autoimmune inflammation [114, 115].

However, despite the development of these sophisticated bioengineering advancements, several notable bottlenecks must be carefully addressed before these strategies can be successfully transitioned from the bench to the bedside, particularly in the management of chronic autoimmune diseases. First, receptor and tissue specificity remain equally important challenges. While the use of paralog-specific monoclonal antibodies can avoid the induction of pan-Notch systemic toxicity, the ubiquitous expression of individual Notch receptors across various nonimmune-related tissues means that secondary, on-target toxicity remains a distinct possibility of long-term administration. Second, the complexity of the autoimmune inflammatory microenvironment complicates tissue-targeted delivery; unlike the leaky vasculature and relatively high permeability and retention effect typical of solid tumors, the various injured tissues in autoimmune diseases exhibit different vascular structures and permeabilities, which may severely compromise the targeting efficiency of customized nanoparticles or nanoprodrugs. Given that autoimmune therapies generally require prolonged or even lifelong management rather than the aggressive, short-term regimens used in oncology, the threshold for acceptable toxicity in the context of the former is exceptionally low: Any subtle, unexpected disturbance in physiological homeostasis could lead to clinical failure, as historically evidenced by the discontinuation of early-generation therapeutics. Therefore, future clinical translation will rely not only on conceptual precision but also on rigorous, long-term safety evaluations of these targeted modalities in nononcology settings.

## Conclusions and perspectives

In the current research landscape, the study of the Notch signaling pathway in the context of autoimmune diseases is transitioning from simple phenotypic descriptions toward a deep understanding of its complex signaling cross-talk. In recent decades, numerous studies have successfully identified the presence of Notch components across various pathogenic cell types, confirming the crucial role of the entire signaling pathway in regulating immune dysregulation and tissue inflammation. However, autoimmune pathogenesis is not driven by individual pathways but rather regulated by intricate interactions between the Notch pathway and other canonical immunoregulatory networks. Future research must prioritize elucidating this multidimensional cross-talk to fully understand progression of autoimmune diseases.

The literature has comprehensively suggested that the Notch pathway plays multiple roles in autoimmune diseases through both human clinical observations and experimental models. The evidence reflects a distinct structural framework, and while clinical data are predominantly observational—relying on genetic polymorphisms, epigenetic methylation, and expression profiling in peripheral blood or target tissues—the underlying mechanisms have been substantiated by diverse in vivo animal models and in vitro cell culture experiments. This multilayered evidence significantly bolsters the biological reasonability of the involvement of Notch-mediated regulatory processes in autoimmune pathogenesis. However, obvious translational limitations remain within the current evidence base. The clinical findings remain largely correlative, and longitudinal validation regarding disease progression or therapeutic response is lacking. Experimental models such as CIA or acute induction methods involve transient inflammatory insults as well as highly controlled in vitro cultures and inherently fail to fully recapitulate the highly heterogeneous and relapsing-remitting chronic pathological characteristic of human autoimmune diseases. Future research must address these gaps across multiple dimensions. In clinical observation studies, the paradigm must shift from cross-sectional analyses toward longitudinal, prospective clinical cohorts for dynamically tracking the molecular changes in the Notch pathway and correlating them with disease activity indices and curative effects. Preclinically, increasing the translational evidence grade is important; this will involve moving beyond descriptive correlations toward multitiered verification of causal relationships through multicenter, independent replication and rigorous randomization and blinding protocols to minimize experimental bias and ensure robust, reproducible conclusions.

Ultimately, this reliance on preclinical models, combined with the widespread expression of Notch receptors in healthy tissues, significantly complicates clinical translation. Clinical data on traditional pan-Notch inhibitors are almost exclusively derived from oncology trials, where nonselective blockade has inevitably triggered prohibitive systemic toxicity, such as intestinal goblet cell hyperplasia. To overcome this severe systemic toxicity, recent translational advancements have emphasized precise and localized targeting strategies. Emerging approaches, including paralog-selective monoclonal antibodies, site-activated prodrugs, and bioengineered delivery platforms, offer promising foundations for safely reprogramming pathogenic immune cells while minimizing side effects. Consequently, next-generation Notch signaling pathway interventions will require bespoke preclinical models and rigorous autoimmune-specific clinical trials to definitively demonstrate their safety and efficacy.

## Data Availability

No datasets were generated or analysed during the current study.
